# Local and Regional Re-Establishment of Cellular Immunity during Curative Antibiotherapy of Murine *Mycobacterium ulcerans* Infection

**DOI:** 10.1371/journal.pone.0032740

**Published:** 2012-02-29

**Authors:** Teresa G. Martins, José B. Gama, Alexandra G. Fraga, Margarida Saraiva, Manuel T. Silva, António G. Castro, Jorge Pedrosa

**Affiliations:** 1 Life and Health Sciences Research Institute (ICVS), School of Health Sciences, University of Minho, Braga, Portugal; 2 ICVS/3B's - PT Government Associate Laboratory, Braga/Guimarães, Portugal; 3 Institute for Molecular and Cell Biology, Porto, Portugal; Institut de Pharmacologie et de Biologie Structurale, France

## Abstract

**Background:**

Buruli ulcer (BU) is a neglected necrotizing disease of the skin, subcutaneous tissue and bone, caused by *Mycobacterium ulcerans*. BU pathogenesis is associated with mycolactone, a lipidic exotoxin with cytotoxic and immunosuppressive properties. Since 2004, the World Health Organization recommends the treatment of BU with a combination of rifampicin and streptomycin (RS). Histological analysis of human tissue samples suggests that such antibiotic treatment reverses the mycolactone-induced local immunosuppression, leading to increased inflammatory infiltrations and phagocytosis of bacilli.

**Methodology/Principal Findings:**

We used a mouse model of *M. ulcerans* footpad infection, followed by combined RS treatment. Time-lapsed analyses of macroscopic lesions, bacterial burdens, histology and immunohistochemistry were performed in footpads. We also performed CFU counts, histology and immunohistochemistry in the popliteal draining lymph nodes (DLN). We observed a shift in the cellular infiltrates from a predominantly neutrophilic/macrophagic to a lymphocytic/macrophagic profile in the infected footpads of antibiotic-treated mice. This shift occurred before the elimination of viable *M. ulcerans* organisms, which were ultimately eradicated as demonstrated by the administration of dexamethasone. This reduction of bacillary loads was accompanied by an increased expression of inducible nitric oxide synthase (NOS2 or iNOS). Predominantly mononuclear infiltrates persisted in the footpads during and after treatment, coincident with the long persistence of non-viable poorly stained acid-fast bacilli (AFB). We additionally observed that antibiotherapy prevented DLN destruction and lymphocyte depletion, which occurs during untreated experimental infections.

**Conclusions/Significance:**

Early RS treatment of *M. ulcerans* mouse footpad infections results in the rapid elimination of viable bacilli with pathogen eradication. However, non-viable AFB persisted for several months after lesion sterilization. This RS regimen prevented DLN destruction, allowing the rapid re-establishment of local and regional cell mediated immune responses associated with macrophage activation. Therefore it is likely that this re-establishment of protective cellular immunity synergizes with antibiotherapy.

## Introduction

Buruli ulcer (BU), caused by the environmental pathogen *Mycobacterium ulcerans*, is a necrotizing disease of the skin, subcutaneous tissue and bone. This disease is the third most common mycobacterial infection in the world, after tuberculosis and leprosy. There has been a growing incidence of BU over the last decade mainly in West African countries, which contributes for the importance of this infection as an emerging infectious disease [1], [2]. The pathogenesis of BU is associated with mycolactone, a lipidic exotoxin produced by *M. ulcerans* that has cytotoxic and immunosuppressive properties [3]–[7].

Until recently, surgical excision of lesions and skin grafting was the only available treatment for BU patients, but recurrence rates varied from 6 to 47% [8]–[10]. In addition, surgery is not reliable in rural poor regions, where the disease is endemic, due to lack of medical care, high costs and prolonged hospitalization [2]. Since 2004, the World Health Organization (WHO) recommends a combination of the antibiotics rifampicin and streptomycin (RS) for the treatment of BU [11]. This recommendation was based on the successful results obtained in a small clinical trial carried on from 2001 to 2002 in Ghana with patients with early non-ulcerative lesions [12], and following promising studies showing bacterial killing in *M. ulcerans*–infected mice treated with rifampicin and an aminoglycoside (amikacin or streptomycin) [13]–[15]. Subsequent clinical trials with higher numbers of patients established the efficacy of the treatment in curing early lesions and arresting or reducing the surface area of more advanced ones often only requiring minimal surgery [16], [17]. A recent study reports healing in 95% of the patients with all forms of the disease treated with RS without surgery, although a long time of therapy is needed for the healing of some advanced cases [18]. The fact that the RS treatment protocol still presents several limitations justifies the need of furthering our understanding on the mechanisms involved in *M. ulcerans* control, both in treated patients as well as during the natural progression of the disease.

Histological features of advanced experimental and BU lesions are characterized by extensive necrotic, acellular areas with clumps of extracellular bacilli surrounded by a band of inflammatory infiltrates composed mainly by neutrophils and macrophages, some with intramacrophage bacteria [19], [20]. In addition, it was recently reported that during mouse footpad infections with virulent *M. ulcerans*, pathogen-specific gamma interferon (IFN-γ)-producing T cells develop early in the draining lymph node (DLN) and that CD4^+^ T cells migrate to the infection foci [21]. However, the progression of infection is accompanied by the local depletion of recruited cells; moreover, there is bacillary dissemination to the DLN accompanied by mycolactone-induced extensive apoptotic cytopathology, leading to depletion of CD4^+^ T cells and abrogation of IFN-γ expression [21] and/or activity [7]. This local and regional immunosuppression compromises the maintenance of the early initiated cellular mediated immunity (CMI) and allows the progression of the disease in susceptible hosts [21]. On the other hand, histological analysis of skin samples from BU patients after completion of successful antibiotic treatment has suggested a reversion of that local immunosuppression, based on the observation of abundant mononuclear infiltrates, including organized lymphoid structures (granulomas and lymphoid clusters near vessels) at the infectious focus [22]. It has been further suggested that these inflammatory alterations occur early after the beginning of antibiotherapy, being accompanied by phagocytosis of bacilli by macrophages [23]. Also, when blood cells of patients treated with antibiotics are stimulated, a higher IFN-γ production is observed [24]. This inflammatory and cytokine type of immune response is characteristic of CMI and delayed-type hypersensitivity (DTH) that are associated with resistance to *M. ulcerans* infection and with spontaneous healing at later stages of the disease (reviewed in [25]).

Altogether, these observations in human studies suggest that a CMI response would associate with the activity of the antibiotics [22], [23]. However, since in the previous studies no direct correlations were addressed between alterations in histology and bacterial viability in the same subjects, it remains unclear whether the immune recuperation associated with efficient antibiotherapy begins before or after the elimination of viable bacilli, which depends on the timing of the host response not only in the infection focus but also in the DLN.

In addition, persistence of acid fast bacilli (AFB) in the lesions has been reported after the end of the treatment period, in both mice and in humans [12], [14], [22], [23], [26], [27], raising the question of *M. ulcerans* being dead or only in a state of latency, as reported for *Mycobacterium tuberculosis* [28], [29]. This may have implications for our understanding on the worsening of lesions or the appearance of new lesions reported in RS-treated patients during or after treatment, the so-called paradoxical reactions, that may be triggered either by *M. ulcerans* antigens or by viable organisms [18], [27], [30]–[32].

We have therefore studied in the mouse footpad model of *M. ulcerans* infection, during and after a RS regimen, the progression of the infection, viability and eradication of bacteria, as well as the dynamics of the cellular host immune responses in both the footpad and the DLN.

## Materials and Methods

### Ethics Statement

This study was approved by the Portuguese national authority for animal experimentation Direcção Geral de Veterinária (ID: DGV 594 from 1^st^ June 2010). Animals were kept and handled in accordance with the guidelines for the care and handling of laboratory animals in the Directive 2010/63/EU of the European Parliament and of the Council.

### Animals

Eight-week-old female Balb/c mice were obtained from Charles River (Barcelona, Spain) and were housed under specific-pathogen-free conditions with food and water *ad libitum*.

### *M. ulcerans* experimental infection

*M. ulcerans* 98–912 (Institute of Tropical Medicine (ITM) collection, Antwerp, Belgium), a mycolactone D producing strain, was isolated in China from a case of ulcer and is highly virulent for mice, as previously described [6], [7], [20]. Preparation of the inoculum was performed as previously described [21]. Mice were inoculated in the left hind footpad with 0.03 ml of *M. ulcerans* suspension containing 5.5 log_10_ AFB. The right hind footpad was used as a control.

### Treatment of mice

Rifampicin and streptomycin were obtained from Sigma-Aldrich (USA). The dose and mode of administration were adapted from previous studies that used a mouse model of *M. ulcerans* infection [33], [34]. Briefly, rifampicin was given orally by gavage at a dosage of 10 mg/kg of body weight. Streptomycin was given by subcutaneous injection, at a dosage of 150 mg/kg of body weight. The treatment was initiated at the second week post-infection when the footpads of mice were swollen to 2.5 mm and was performed 6 days per week during 10 weeks. Antibiotic vehicles were given to control mice.

### Immunosuppressive treatment

Dexamethasone (Sigma-Aldrich) administration was adapted from a previous study that used a mouse model of reactivation of latent *M. tuberculosis* [29]. Briefly, dexamethasone was given by intraperitoneal injection at a dosage of 5 mg/kg of body weight. The administration was initiated at week 9 after the end of antibiotic treatment, and given 6 days per week for 10 weeks. Dexamethasone vehicle was given to control antibiotic treated mice.

### Assessment of footpad swelling and bacterial growth

After infection, as an index of lesion development, footpad swelling of infected mice was determined over time, as previously described [20]. The non-treated mice were sacrificed after the emergence of ulceration at 24 days post-infection, and no further parameters were evaluated for this group. *M. ulcerans* growth in footpad tissues of infected mice and in popliteal DLN that drains the infected footpad, was evaluated by colony forming units (CFU) and AFB counts at 15, 24, 42, 81, 143 and 217 days post-infection. Footpads were homogenized as previously described [20]. At this time, samples for AFB counts were collected and determined according to the method described by Shepard and McRae [35]. Suspensions were decontaminated with hydrochloric acid (HCl) 1 M containing 0.001% phenol red solution for 15 min., followed by neutralization with sterile sodium hydroxide 1 M. Suspensions were centrifuged and ressuspended in PBS. DLN were homogenized and the cell suspensions were lysed with saponin 0.1%. Serial dilutions of the footpad and DLN homogenates were plated on 7H9 agar. CFU's were counted after 6–8 weeks of incubation at 32°C.

### Flow cytometry

Single cell suspensions of the popliteal DLN were stained with a combination of fluorochrome-labeled monoclonal antibodies specific for CD3 (clone 145-2C11, BD Pharmingen, USA) and CD19 (clone 1D3, BD Pharmingen), for 20 min. at 4°C. Cells were fixed overnight with 2% formalin in PBS with 0.5% BSA and 0.01 M azide (Sigma) and analyzed using BD FACSDiva™ version 6 software on a Becton Dickinson LSR II flow cytometer.

### Histological and immunofluorescence studies

Mouse popliteal DLN and footpads were harvested, fixed in buffered formalin and embedded in paraffin. Light-microscopy studies were performed on tissue sections stained with hematoxylin and eosin (HE) or Ziehl Neelsen (ZN), as previously described [20]. The histological analysis of mouse footpads was performed by the identification of the type of predominant inflammatory infiltrate (when present) in each field of 40× objective, attributing the classifications of “predominantly mononuclear” or “predominantly “neutrophilic”. The relative abundance of inflammatory cells in the infiltrates was classified as scarce (1), intermediate (2) or abundant (3) using a 40× objective. The distribution of AFB was classified as “predominantly intracellular” or “predominantly extracellular”, depending on the localization of the majority of bacteria in each field, using a 100× objective.

For immunofluorescence staining, tissue sections were deparaffinised and hydrated. Antigen retrieval was performed with Citrate Buffer (Lab Vision Corporation, USA) for 20 min. For the detection of germinal centers in DLN, FITC fluorochrome-labeled *peanut agglutinin* (PNA) (Sigma) was added to the sections at a concentration of 1∶100 for 2 h at room temperature. For the detection of the enzyme inducible nitric oxide synthase (NOS2) in the footpads, tissues were incubated with purified rabbit polyclonal NOS2 (clone M-19) (Santa Cruz Biotechnology, USA) at a concentration of 1∶100 followed by overnight incubation at 4°C. Goat anti-rabbit AlexaFluor 568 secondary antibody (Molecular Probes, USA) was added at a concentration of 1∶500 for 1 h at room temperature. Afterwards, for the detection of *M. ulcerans*, NOS2 stained sections were incubated with Auramine-O (Polysciences, Inc., USA) for 30 min. at room temperature and decolorized with 0.5% HCl fuming 37% in absolute ethanol. For all sections, DAPI (4¢,6-diamido-2-phenylindole hydrochloride, Molecular Probes) was used to detect nuclei. Images were obtained with an Olympus BX61 microscope.

### Statistical analysis

Differences between the means of experimental groups were analyzed with the two-tailed Student's *t* test, with a 95% level of significance, using the GraphPad Prism version 5.0 software. For percentage values, the statistical analysis was performed after transforming them to arcsine values. Differences with a *P* value<0.05 were considered significant.

## Results

### Highly virulent *M. ulcerans* is eradicated from the footpad following RS treatment while non-viable bacilli persist for several months

**Figure 1:**
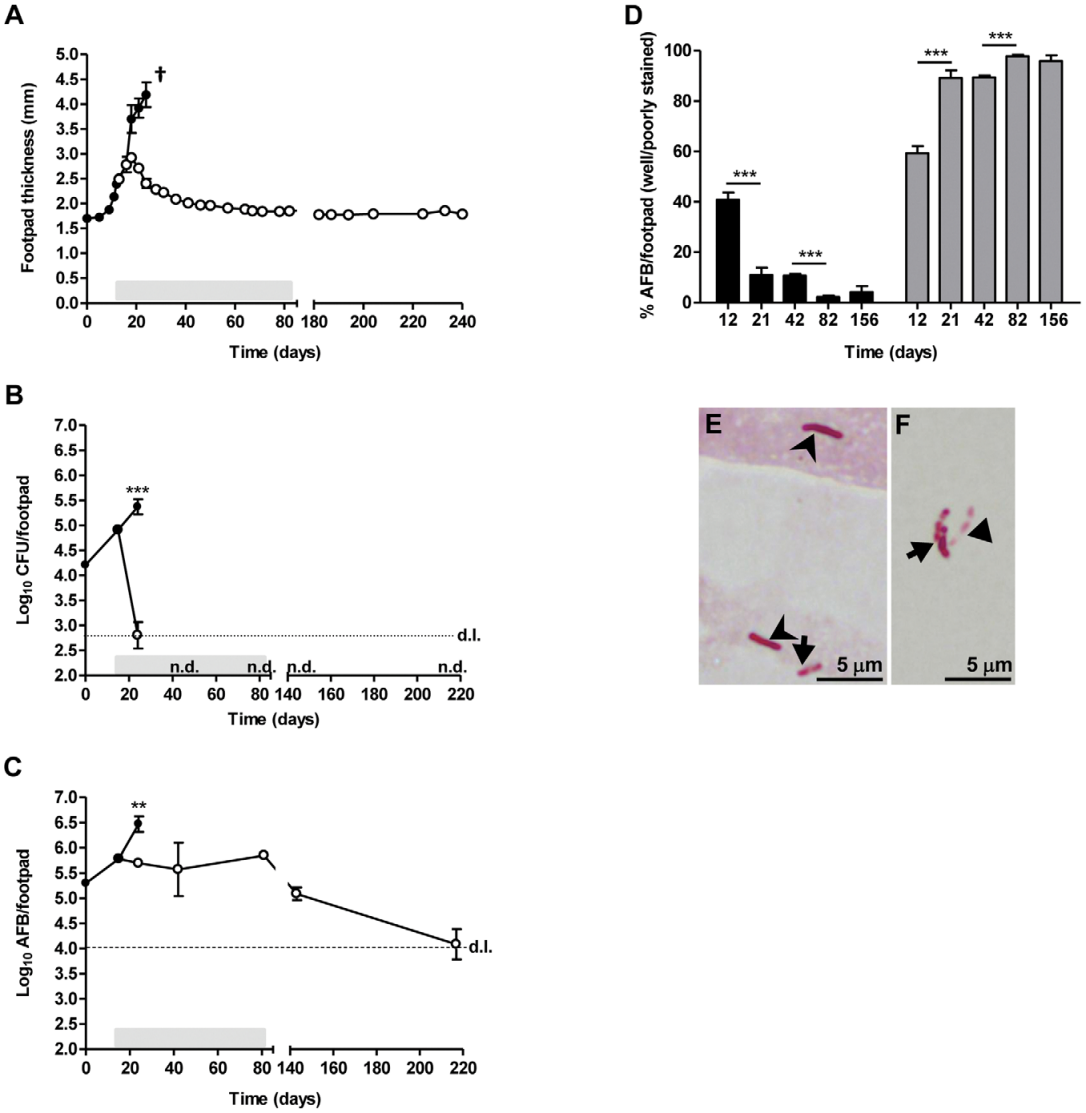
Lesion progression and bacterial proliferation of *M. ulcerans* in the footpads of mice treated or non-treated with RS. Mice were infected with *M. ulcerans* strain 98–912 and were left untreated (closed circles) or subjected to treatment with RS for 10 weeks (open circles). (A) Lesion progression was assessed by measurement of footpad swelling (n = 15). Bacterial proliferation was assessed by CFU (B) and AFB counts (C) (n = 5). (D) Percentage of well stained (black bars) versus poorly stained (including beaded) (grey bars) AFB in the footpad for the antibiotic treated group of mice. At day 12 post-infection, treatment was initiated until day 82. (E) AFB at day 12 post-infection, with well stained (arrowheads) and beaded (arrow) bacilli. (F) AFB at day 156 post-infection, with beaded (arrow) and poorly stained (triangle) bacilli. Asterisks represent significant differences between treated and non-treated mice on panels B and C (**, *P*<0.01, ***, *P*<0.001). Significant differences over time for the well stained or poorly stained group in panel D were determined by comparing each time point with the following (***, *P*<0.001). Grey bar on panels A, B and C represents the time period of antibiotic administration. d.l., detection limit; n.d., not detected. †, mice were sacrificed for ethical reasons after the emergence of ulceration. Results are from one representative experiment of two independent experiments. Data points and bars represent the mean ± SEM.

We subcutaneously infected mouse footpads with 5.5 log_10_ AFB of the highly virulent *M. ulcerans* strain 98–912 and started antibiotic treatment at the second week post-infection, when footpad swelling had reached 2.5 mm (Figure 1A) and bacterial growth 4.91 log_10_ CFU's (Figure 1B). In non-treated mice we observed progression of footpad swelling to around 4.2 mm in 4 weeks (Figure 1A), time point at which mice started showing signs of ulceration, while in treated mice the progression of swelling was halted after only one week of RS treatment (Figure 1A). Furthermore, we observed a progressive reduction of footpad swelling until normal values by the end of the treatment period, which was maintained until the end of the experimental period, 5 months later (Figure 1A). The reduction of footpad lesions correlated with a significant decrease in CFU counts (*P*<0.001), observed as early as two weeks of treatment (Figure 1B). By 42 days post-infection and until the end of the experimental period (82, 143 and 217 days post-infection) CFU counts were undetectable (Figure 1B).

Interestingly, comparative analysis of CFU and AFB values (Figure 1B and 1C) showed that non-culturable bacilli identified by AFB ZN staining only started to decline by the end of the treatment period (82 days post-infection) and persisted for several months in the footpad tissue (Figure 1C). Indeed, 9 and 20 weeks after the end of treatment (143 and 217 days post-infection, respectively), we still found AFB in treated mice (Figure 1C). However, while in non-treated mice the acid-fastness (as assessed by ZN) of approximately half of bacilli remained normal throughout infection (data not shown), in antibiotic-treated animals the acid-fastness faded over time, with the predominance of beaded and poorly stained (ghost) bacilli (*P*<0.001 from 12 to 21 days and from 42 to 82 days post-infection) (Figure 1 D and F).

To assess if the AFB persisting in the footpad tissue of treated mice corresponded to dead bacteria, we used an adapted protocol of the Cornell model [29]. Corticosteroid administration was performed during 10 weeks, starting at week 9 after the end of RS treatment. In contrast to what happens in the *M. tuberculosis* model of latency [28], [29], we did not observe reactivation of *M. ulcerans* infection as viable bacilli were not detected, despite the persistence of AFB (Table 1).

**Table 1:** Dexamethasone administration did not reactivate *M. ulcerans* infection in mice previously treated with antibiotics.

Day of DEX^1^ treatment	Group	CFU	AFB
0	Control	Not detected	5.09 log_10_±0,25
74	Control	Not detected	4.09 log_10_±0,61
	DEX	Not detected	4.07 log_10_±0,64

Antibiotic treated mice were left for a period of 8 weeks, and then subjected to dexamethasone^1^ administration during 10 weeks (74 days). Bacterial load was determined by CFU and AFB counts at the beginning and at the end of administration. Values are mean ± standard deviation.

### Antibiotic treatment is associated with a re-establishment and maintenance of the early-developed local mononuclear inflammatory response to *M. ulcerans*

**Figure 2:**
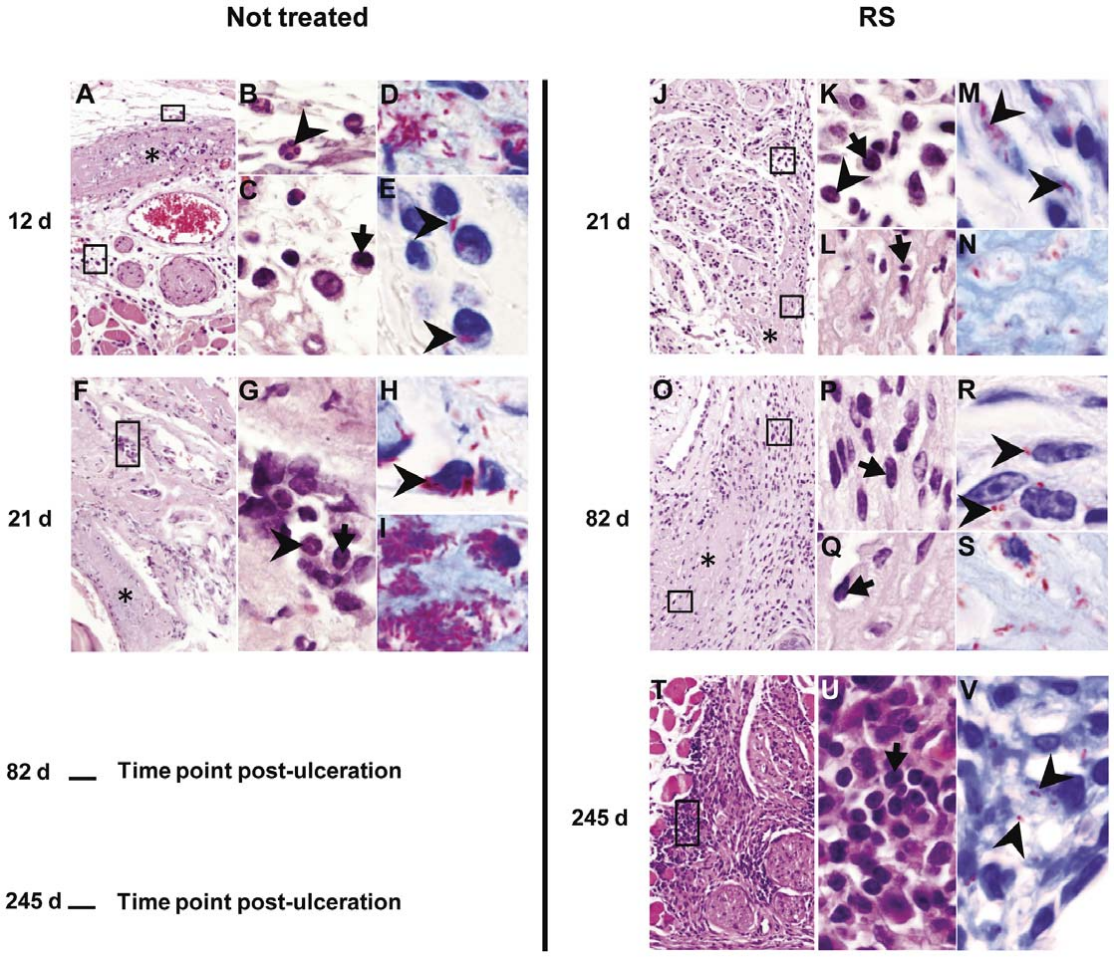
Histology of mice footpads infected with *M. ulcerans* and treated or non-treated with RS. Histological sections of footpads collected at different time points were stained with HE (A, B, C, F, G, J, K, L, O, P, Q, T and U) or ZN (D, E, H, I, M, N, R, S and V). Magnifications: ×10 (A, F, J, O and T), ×60 (B, C, G, K, L, P, Q and U) and ×100 (D, E, H, I, M, N, R, S and V). At day 12 post-infection, treatment was initiated until day 82. (A and F) Footpads of non-treated mice (at 12 and 21 days post-infection, respectively) with necrotic areas (asterisks). Magnifications of panel A and F (rectangles) show neutrophils (B and G; arrowheads) and mononuclear cells (C and G; arrows) at the periphery of necrotic areas. (D and I) Clusters of extracellular bacilli in necrotic areas. Intracellular bacilli (E; arrowheads), and intra (H; arrowhead) and extracellular bacilli (H) are observed at more distant regions from necrotic areas at 12 and 21 days post-infection, respectively. (J, O and T) Footpads of antibiotic treated group of mice at 21, 82 and 245 days post-infection show abundant cellular infiltration, composed mainly by mononuclear cells (K, P and U; arrows), and few scattered neutrophils (K; arrowhead). Necrotic areas are reduced in panel J and O (asterisk). Magnifications of those areas (rectangles) show the presence of mononuclear cells (L and Q, arrows). A predominance of extracellular bacilli is found in those areas (N and S), and intracellular bacilli are present at more distant regions (M and R; arrowheads). (V) Tissue shows few, scattered bacilli (arrowheads) in areas of cellular infiltration. Results are from one representative experiment of two independent experiments.

**Figure 3:**
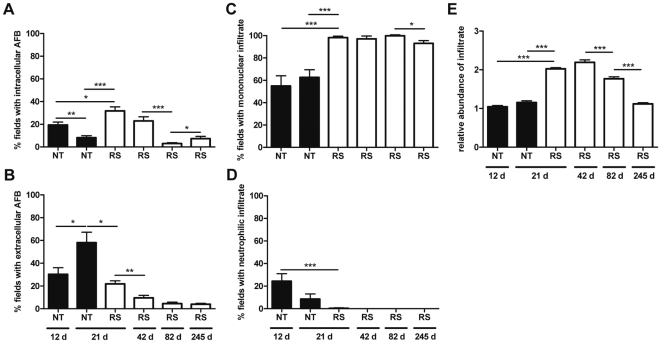
Histological analysis of mice footpads infected with *M. ulcerans* and treated or non-treated with RS. Histological sections of footpads collected at different time points post-infection were stained with HE or ZN and the percentage of fields with AFB localized predominantly inside (A) or outside (B) cells and the percentage of fields with predominance of mononuclear (C) or neutrophilic cells (D) was evaluated. (E) Relative abundance of inflammatory infiltrates. Analyses were performed in 6 different sections of each sample of mice footpad, in a total of 3 footpads per group. Asterisks represent significant differences between treated and non-treated mice at 21 days post-infection (*, *P*<0.05; ***, *P*<0.001). Significant differences over time for the non-treated or RS group were determined by comparing each time point with the following (*, *P*<0.05; **, *P*<0.01, ***, *P*<0.001). NT; non-treated mice. RS; mice treated with rifampicin and streptomycin. Time points represented are days post-infection. Treatment duration was as described in Figure 2 legend. Data bars represent the mean ± SEM.

Histopathological analysis showed edematous and necrotic lesions (Figure 2A) already established in the footpad tissue when the RS treatment was initiated. As previously described in *M. ulcerans* progressing lesions from humans and mice [19], [20], necrosis was surrounded by an inflammatory infiltrate composed mainly by macrophages and neutrophils (Figure 2A, 2B, 2C, 3C and 3D). These necrotic areas contained clumps of extracellular bacilli (Figure 2D), whereas in peripheral infiltrates intracellular bacteria were predominantly seen (Figure 2E). Extensive edema was also evident (Figure 2A and 1A). After starting RS treatment at day 12 post-infection, we observed an increase of inflammatory infiltrates as early as week 3 post-infection (Figure 2J and 3E; *P*<0.001), accompanied by a switch to a profile with a predominance of lymphocytes and macrophages (Figure 2K, 3C and 3D; *P*<0.001). Despite the persistence of necrotic areas (Figure 2J) with some extracellular bacilli (Figure 2N), mononuclear cells were now present in these areas (Figure 2L). We also observed a higher percentage of areas with intracellular bacilli (Figure 3A; *P*<0.05), which were mainly found in the peripheries of the remaining necrotic tissue (Figure 2M). In contrast, at the same time point, non-treated mice showed an increase of tissue areas with clumps of extracellular bacilli (Figure 2I and 3B; *P*<0.05), correlating with the expansion of necrotic areas and the emergence of footpad ulceration (Figure 2F). Nevertheless, some peripheral phagocyte-rich infiltrates could still be found (Figure 2G and 2H).

At the last week of RS treatment, footpad tissues were almost devoid of necrotic areas (Figure 2O), although some histological sections revealed a disorganized tissue, invaded by mononuclear cells (Figure 2Q) and co-localized with extracellular bacilli (Figure 2S). However, the extracellular bacilli, as well as bacteria localized at the peripheral zones with cellular infiltrates (Figure 2R), were poorly stained, which is consistent with the absence of detectable CFU (Figure 1B) and presence of beaded and ghost AFB in the tissue homogenates (Figure 1C, 1D and 1F). In addition, the maintenance of extensive inflammatory infiltrates, mainly composed by mononuclear cells, was observed (Figure 2P, 2Q, 3C and 3E). This infiltrate persisted 5 months after the end of the treatment (Figure 2T, 2U, 3C and 3E), coincident with the persistence, although decreased, of tissue areas containing poorly stained AFB, observed both intra and extracellularly (Figure 2V, 3A and 3B).

### Efficient elimination of *M. ulcerans* by RS treatment is associated with activation of macrophages

**Figure 4:**
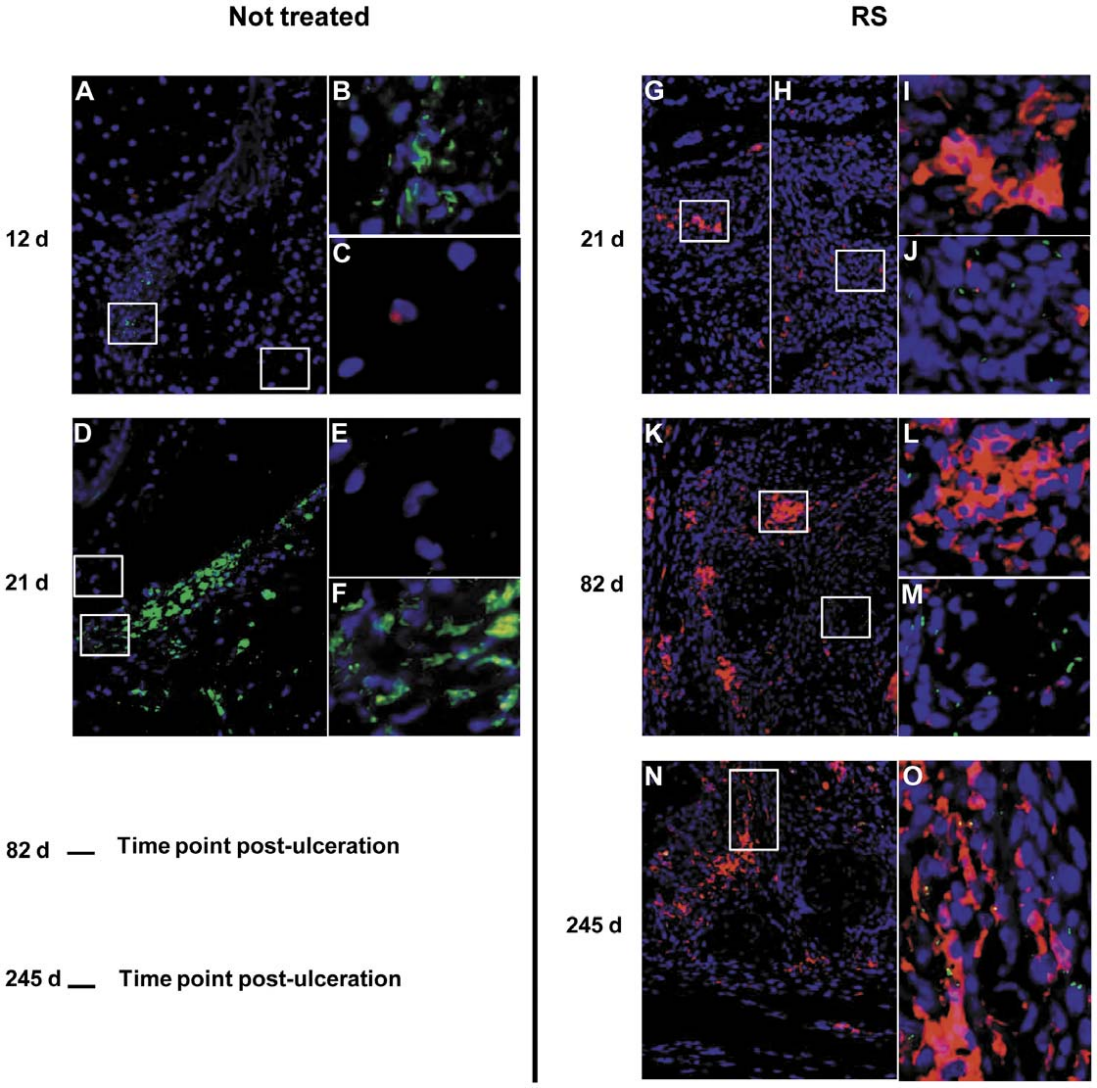
Immunohistochemistry for NOS2 of mice footpads infected with *M. ulcerans* and treated or non-treated with RS. Histological sections of footpads collected at different time points were stained with DAPI (blue; nuclei staining), anti-NOS2 antibody (red) and auramine-O (green; mycobacteria staining). Magnifications ×10 (A, D, G, H, K and N) and ×40 (B, C, E, F, I, J, L, M and O). Treatment duration was as described in Figure 2 legend. (A) Footpads of non-treated mice at 12 days post-infection with no evidence for NOS2 presence in the tissue in areas with numerous bacilli (B, green) and only few cells are stained at peripheries (C). (D) At 21 days post-infection huge clumps of bacilli are observed with no evidence for NOS2 staining (F), which is neither present at the periphery (E). (G and H) Footpads of mice treated with RS at 21 days post-infection show little NOS2 staining in areas co-localized with few (J) or absent bacilli staining (I). (K) Footpads of mice treated with RS at 82 days post-infection show more numerous and larger areas of NOS2 with little co-localization with bacilli (L), whereas areas with numerous extracellular bacilli show little staining for NOS2 (M). (N and O) Footpads of mice treated with RS at 5 months after finalizing the treatment (245 days post-infection) show NOS2 staining co-localized with bacilli. Results are from one representative experiment of two independent experiments.

Phagocytes, in particular macrophages, are the main effector cells involved in mycobacterial phagocytosis and intracellular killing. One of the mechanisms of mycobacterial killing is the production of nitric oxide (NO) in the macrophage phagosome, mediated by the iNOS (or NOS2) [36]–[38]. The expression and production of this enzyme in macrophages is, in turn, largely mediated by the IFN-γ-dependent pathway [37], [39], [40]. It was previously shown that NO production is important for *M. ulcerans* killing within macrophages [7]. Therefore, as a marker of phagocyte activation, we were prompted to evaluate by immunohistochemistry the presence of NOS2 at the site of *M. ulcerans* infection, both during and after antibiotic treatment. Before the beginning of RS treatment, at day 12 post-infection (Figure 4A), we did not find staining for NOS2 in the footpad areas with bacilli clumps (Figure 4B), and, at the periphery of infectious focus, NOS2 staining was very scarce, with only a few positive inflammatory cells (Figure 4C).

At the emergence of ulceration in non-treated mice (day 21 post-infection), larger areas with clumps of bacilli were observed (Figure 4D and F), and, again, NOS2 staining was absent, as well as in the peripheral areas (Figure 4E). On the other hand, at the same time point, RS-treated mice presented footpad tissue with NOS2 staining (Figure 4G and 4H), particularly in areas with few or absent bacilli (Figure 4I), as compared to regions with higher bacilli numbers (Figure 4J). At the end of the treatment period (82 days post-infection) numerous and larger NOS2-positive areas were seen (Figure 4K) in regions with few or absent AFB (Figure 4L), and fewer NOS2-positive cells were found in the areas with increased bacterial load (Figure 4M). Interestingly, 5 months after the end of the treatment (Figure 4N), areas with NOS2 were still present and appeared co-localized with AFB (Figure 4O).

### Early antibiotic treatment prevents DLN destruction induced by colonization with *M. ulcerans*

**Figure 5:**
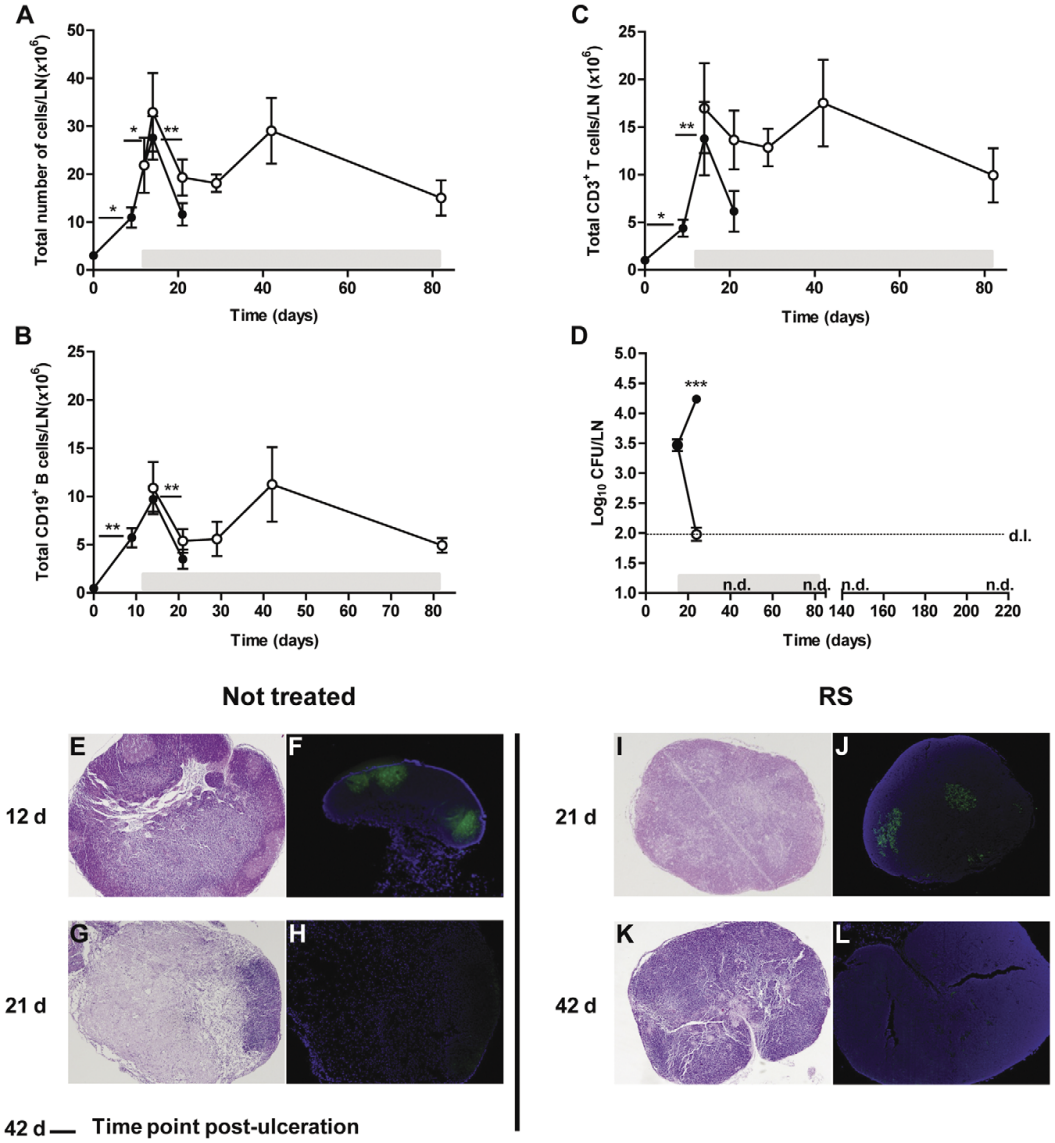
Leukocyte kinetics, bacterial proliferation and DLN histology of mice infected with *M. ulcerans* and treated or non-treated with RS. (A) Total number of leukocytes in the DLN were determined at different time points post-infection in non-treated (closed circles) and antibiotic-treated mice (open circles) (n = 5). (B) Total number of B cells (CD19^+^) and (C) T cells (CD3^+^) in the DLN were determined by flow cytometry (n = 5). (D) Bacterial proliferation was assessed by CFU counts; d.l., detection limit; n.d., not detected. Significant differences between values over time in non-treated or treated mice on panel A, B and C were determined by comparing each time point with the following (*, *P*<0.05; **, *P*<0.01). Significant differences between treated and non-treated mice on panel D (***, *P*<0.001). Grey bar represents the time period of antibiotic administration. Histological sections of DLN collected at different time points were stained with HE (E, G, I and K) or with FITC fluorochrome-labeled PNA (green) counterstained with DAPI (blue; nuclei staining) for the labeling of germinal centers (F, H, J and L). Magnifications ×4. (E and I) DLN of non-treated mice at day 12 and treated mice at day 21 post-infection, respectively, showing enlargement of the DLN with germinal centers (F and J). (G) DLN of non-treated mice at day 21 post-infection showing extensive cell depletion and the absence of germinal centers (H). (K) DLN of treated mice at day 42 post-infection showing a normal structure and absence of germinal centers (L). Results are from one representative experiment of two independent experiments. Data points represent the mean ± SEM.

Lymphocytes recruited to the site of infection are initially differentiated and activated in the DLN; therefore, characterization of adaptive immune responses in this lymphoid organ is essential to disclose host mechanisms of protection. Previous studies from our laboratory showed that the initial increase of the total number of cells, including B and T cells, in the popliteal DLN upon footpad infection by *M. ulcerans* strain 98–912 is followed by a rapid decrease of cell numbers [21]. This profile was confirmed in this study (Figure 5A, 5B and 5C), with a significant increase of the total number of cells from day zero to day 14 (*P*<0.05), followed by a significant decrease in the number of total cells at day 21 post-infection (*P*<0.01). This decline is related to the colonization of DLN by viable bacteria (as observed in Figure 5D) that induce apoptotic cell death associated with the production of mycolactone, as previously described [21].

We now show that antibiotherapy resulted in the prolonged maintenance of cells in the DLN (Figure 5A, 5B and 5C), as we did not observe statistical significant differences over time since the beginning of the treatment, which was correlated with a clearance of CFU counts in the DLN (Figure 5D). Analysis of histopathology 21 days post-infection showed that in non-treated animals, the structure of the DLN was damaged, with the depletion of cells and the absence of organized germinal centers (Figure 5G and 5H) that preceded footpad ulceration, as previously described [21], while in treated animals the structure of the DLN was maintained (Figure 5I and 5J). After 4 weeks of treatment, when viable bacilli were already absent from the footpad (Figure 1B) and from the DLN (Figure 5D), germinal centers were not seen in the histological sections of DLN (Figure 5L), indicating that this lymphoid organ returned to its steady state structure.

## Discussion

Since the recommendation by WHO in 2004 of treating BU patients with a regimen of antibiotherapy combining RS, clinical studies gave rise to the hypothesis that a synergistic effect between antibiotics and the host immune system is in place to clear *M. ulcerans* infection [18], [22], [23]. RS are bactericidal against extracellular and intramacrophage susceptible mycobacteria [41] and the quick reduction in *M. ulcerans* CFU counts in treated mice ([14], [42], [43] and present results) suggests that such mode of antimycobacterial activity also operates *in vivo*. However, the possible participation of the host immune system in the control of the infectious process remains an open question. Considering the limitations of human studies, experimental infections performed in animal models constitute a crucial contribution for a detailed investigation on the host immune response to *M. ulcerans* during antibiotherapy. This approach may well open the possibility of immune-based interventions for the treatment of BU as an alternative or a complement to the current RS protocol. Indeed, the WHO RS protocol presents several limitations, including the insufficient response of advanced lesions [44], the long period of administration of intramuscular streptomycin leading to poor compliance as it demands skilled personnel and is accompanied by adverse side effects (especially ototoxic effects) [17], [18], and the occurrence of paradoxical reactions characterized by the worsening of lesions or the appearance of new lesions [18], [27], [30]–[32]. Additionally, the study of the host immune response to *M. ulcerans* during RS may help advance the knowledge of immune-mediated mechanisms of protection operating in antibiotic-treated hosts.

Using a mouse model of footpad infection with a highly virulent strain of *M. ulcerans*, we herein show that a curative RS regimen is associated with alterations in the histopathological pattern at the infectious focus, suggesting a waning of the mycolactone-dependent immunosuppressive state that follows an early and transient local CMI response [21]. This reversion allows the reinforcement of the host cellular immune response and the activation of macrophages, accompanying the quick reduction in the number of viable bacilli. In addition, RS treatment is associated with the rapid reduction in viable *M. ulcerans* numbers in the DLN and development and maintenance of CMI in this lymphoid organ.

Our observations in the mouse model share features with the histopathological findings reported for BU treated patients [22], [23], although accelerated in time. Indeed, mixed cellular infiltrations with intracellular bacilli surrounding areas of necrosis with extracellular bacilli are also seen in patients' ulcers after 4 weeks of treatment, [23], comparable to what is observed in mouse footpads after 2 weeks of treatment. In treated patients, only at 8 weeks of treatment macrophage/lymphocyte predominant infiltrates and intra and extracellular bacterial debris were reported [22], [23], as is the case of footpads already at 4 and 8 weeks post-treatment. Regarding the descriptions of pre-ulcerative lesions in humans, abundant macrophage/neutrophil infiltrates are predominant at 4 weeks of treatment, with a large proportion of bacteria inside phagocytes [23]. In line with this, our mouse histological analysis suggests that antibiotherapy is associated with a shift in the type of inflammatory infiltrates from neutrophil/macrophage-predominant to macrophage/lymphocyte-predominant, associated with the enhancement of *M. ulcerans* phagocytosis. The fact that the histological alterations associated with RS antibiotherapy are observed earlier in the mouse model may be correlated with the smaller size of lesions. Additionally, the treatment in mice is initiated when the lesions are still in an early stage. Therefore, the antibiotics may reach the core of the lesion easier and faster in mice than in patients.

A previous study showed that an early T cell-mediated immune response is developed in mice upon infection with *M. ulcerans*, with migration of pathogen-specific CD4^+^ T cells from the DLN to the site of infection [21]. However, progressive infection leads to the depletion of these cells by mycolactone-dependent local destruction [21], resulting in persistence of neutrophilic infiltrates in the lesion [20]. Subsequently, T cell depletion by the same mechanism also occurs in the DLN, due to the node colonization by viable mycolactone-producing *M. ulcerans* [21]. Here we show that early RS chemotherapy prevents DLN destruction and depletion of T and B cells, which may allow the maintenance of lymphocyte activation and migration to the site of infection. This may have an important role in the switch of the inflammatory pattern observed in the lesion soon after the beginning of treatment, due to the reversion of the local immunosuppression by the antibiotic-induced killing of mycobacteria, which, in turn allows the recruitment and survival of activated lymphocytes in the footpad, and the re-establishment of a local CMI. The data regarding the presence of NOS2 only in treated footpads support this interpretation. As previously described [20], [21], [25], CMI responses are protective against *M. ulcerans* infections, as is the case with other mycobacteria, and IFN-γ was found to activate macrophages, through phagolysosome fusion and NO production, with the boosting of their activity against *M. ulcerans*; an effect that is abrogated by mycolactone [7].

Comparing CFU and AFB counts for non-treated mice, we conclude that in untreated lesions numerous *M. ulcerans* bacilli visible by microscopy are non-culturable, an observation that is in accordance with previous reports on mice and humans infected with *M. ulcerans* [12], [15], [26] or *Mycobacterium leprae* [45]. As previously reported in studies of both murine and human *M. ulcerans* infections [12], [14], [15], [33], the antibiotherapy-associated decline in cultivable bacilli is not paralleled by a corresponding decline in the number of AFB; number that in our study was maintained during the 10 weeks of treatment, before starting to decrease. In the case of *M. tuberculosis* infection, reports in mice suggest that non-culturable bacilli, observed during and after treatment are not dead, but rather in a state of latency, as spontaneous reactivation may occur or by corticosteroid administration [28], [29], [46], [47]. However, in our model, the fact that the *M. ulcerans* bacilli have an altered morphology suggests degradation after bacterial killing, as also reported in humans [22], [23], [32]. This hypothesis was confirmed by the non-reactivation of infection after treatment, including after corticosteroid administration, which demonstrates that bacilli are effectively dead. The persistence of AFB thus indicate that the degradation and complete clearance of dead bacteria by macrophages is largely delayed, which may be due to the difficulty of incoming phagocytes to access the necrotic areas where most extracellular bacilli accumulate. Alternatively, sufficient amounts of mycolactone could be still present at these sites [48], inhibiting the activity of phagocytes. This hypothesis is also supported by the low presence of NOS2 at the sites of higher bacillary content. In addition, the persistence of AFB further indicates that the assessment of antibiotherapy efficacy by AFB counts is inadequate, as previously suggested [14], [33]. On the other hand, the prolonged permanence of AFB in tissues explains the persistence of inflammatory infiltrates with activated NOS2-producing phagocytes, found more than one year after the end of the treatment (data not shown).

Although we did not find evidence for pathological effects associated with this prolonged inflammatory response in our mouse model, neither by macroscopic or histological analysis, our findings are in line with the observations of immune-mediated paradoxical reactions in humans, characterized by the worsening of treated lesions or by the appearance of new lesions during or after treatment. Indeed, these pathological reactions in patients submitted to RS chemotherapy are histologically characterized by an abundant inflammatory response and degraded bacteria [27], [30], [32]. Therefore, experimental studies should be developed in animal models to modulate paradoxical reactions in order to clarify if a down-regulation of this immune response or the surgical removal of the lesion core may control this type of reaction.

In summary, we conclude that the early treatment of murine *M. ulcerans* infection with an antibiotic regimen adapted from the WHO protocol is responsible for the waning of the local/regional state of immunosuppression induced by mycolactone, which is observed before the complete clearance of viable bacilli. This further suggests that the rapid re-establishment of the host immune response, through the rescue of DLN function, may participate in the clearance of infection and/or in the resolution of infection-associated histopathology. We also found that poorly stained AFB persist for many months after antibiotherapy at the infection focus and that these bacilli are dead, although capable of inducing a long-term inflammatory response. These observations give insights on the inflammatory nature of the paradoxical reactions to RS treatment recently described in BU patients [18], [27], [30]–[32]. Future studies are needed for the improvement of the available non-surgical therapeutical approaches against BU, which should target not only antimicrobial activity but also immunomodulation, aiming at potentiating bactericidal activity as well as controlling the exacerbated inflammatory responses.
